# Chronic inflammatory demyelinating polyneuropathy with anti-contactin-associated protein 1 antibody and bile duct hamartomas in the liver: a case report

**DOI:** 10.1186/s13256-022-03277-y

**Published:** 2022-02-10

**Authors:** Shurong Hu, Yin Hu, Qiang Du

**Affiliations:** 1 Department of Gastroenterology, The Second Affiliated Hospital, Zhejiang University School of Medicine, Hangzhou, Zhejiang China; 2 Department of Neurology, The Second Affiliated Hospital, Zhejiang University School of Medicine, 88 Jiefang Road, Hangzhou, 310000 Zhejiang China

**Keywords:** Chronic inflammatory demyelinating polyradiculoneuropathy, Autoantibody, Contactin-associated protein 1, Bile duct hamartomas in liver

## Abstract

**Background:**

Autoantibodies targeting node of Ranvier proteins are rarely reported in China.

**Case presentation:**

We present the case of a 66-year-old Chinese man who concomitantly developed chronic inflammatory demyelinating polyneuropathy with anti-contactin-associated protein 1 antibody and bile duct hamartomas in liver, which are rarely reported in China. The man presented with chronic progressive sensory and motor symptoms, bilateral periphery facial paralysis, and protein–cell dissociation of cerebrospinal fluid. Nerve conduction study indicated demyelinating neuropathy. Enhanced magnetic resonance imaging of the liver showed diffuse intrahepatic lesions, which were considered as bile duct hamartomas in the liver. He was suspected as having chronic inflammatory demyelinating polyneuropathy and treated with intravenous immunoglobulin and prednisone. However, his condition got worse. One month later, he was diagnosed with chronic inflammatory demyelinating polyneuropathy associated with anti-contactin-associated protein 1 antibody. He received high-dose methylprednisolone, followed by standard plasma exchange and rituximab therapy. His sensory and motor manifestations were significantly improved at 1 year of follow-up.

**Conclusions:**

This case reminds clinicians to be aware of antiparanodal antibodies, which are associated with specific phenotypes and therapeutic response.

## Background

Chronic inflammatory demyelinating polyneuropathy (CIDP) is a heterogeneous chronic autoimmune neuropathy characterized by chronic progressive motor and sensory deficits during a period of at least 8 weeks [1, 2]. CIDP diagnosis is based on clinical and electrophysiological criteria [2]. Humoral factors are involved in the mechanisms. Intravenous immunoglobulin has been established as the first-choice treatment for CIDP. Other treatments, including corticosteroid and plasma exchange are also applicable [3]. In recent years, autoantibodies targeting node of Ranvier proteins such as contactin-1 (CNTN1), contactin-2 (CNTN2), contactin-associated protein 1 (Caspr1), neurofascin 155 (NF155), and neurofascin 186 (NF186) have been detected in a small subset of patients with CIDP, which disrupt axoglial junctions at nodes or paranodes [4–6]. However, these antibodies against the node of Ranvier proteins are rare [7]. To our knowledge, no cases of CIDP with anti-Caspr1 have been previously reported in Chinese patients.

## Case presentation

We present the case of a 66-year-old Chinese male patient with no previous relevant history or family history of neurological disease. He initially noticed numbness of the feet and weakness of the legs. Two months later, the numbness spread to his thigh root, followed by weakness and numbness of his hands. Subsequently, he presented with gait ataxia and distal limb numbness. Therefore, he went to a local hospital for help. He showed mild facial bilateral paralysis. As his symptoms worsened, it was difficult for him to walk without assistance. Clinical examination showed that muscle weakness and sensory disturbance were observed in the distal parts of all limbs. In a manual muscle strength test, his upper limb muscle strength was grade 4 and lower limb muscle strength was grade 3. Deep tendon reflexes had disappeared in all limbs and no muscle atrophy was observed. No other abnormalities were found in the patient’s cranial nerves or autonomic nervous system. A head computerized tomography (CT)-scan was normal. He then underwent a lumbar puncture, which revealed albuminocytological dissociation, with protein concentration of 2.78 g/L and 2 cells /μl. Electromyography showed that sensory nerve conduction was prolonged in the upper and lower limbs, while motor nerve conduction and F-wave latency was prolonged in the upper limbs and disappeared in the lower limbs. Lumbar spine magnetic resonance imaging (MRI) showed mild herniated disc at lumbar segments 4 and 5 (L4–L5) and lumbar segment 5 and sacral segment 1 (L5–S1). Cervical spine MRI showed mild herniated disc at cervical segment 3–6 (C3–C6). The thorax–abdomen–pelvis CT scan detected no malignancy. A biochemical examination revealed no abnormal findings, with the exception of low hemoglobin (124 g/L), low albumin (30.4 g/L), high cholesterol (7.13 mmol/L), and high D-Dimer (1.48 mg/l). The results of screening tests for human immunodeficiency virus, syphilis reaction, antineutrophil cytoplasmic antibody (ANCA), antinuclear antibody (ANA), common cancer markers, and lead level were negative. The patient’s levels of creatine kinase and vitamins B1, B12, and E were normal. Other normal or negative antibody tests included myelin basic protein (MBP), myelin oligodendrocyte glycoprotein (MOG), aquaporin-4 (AQP4), antibodies associated with paraneoplastic neurological syndromes (PNS), and antiganglioside complex antibody profiles. He was suspected as CIDP and treated with intravenous immunoglobulin (0.4 g/kg/day for five consecutive days) and prednisone tablets 40 mg daily. The symptoms got worse after 1 month of therapy.

**Fig. 1:**
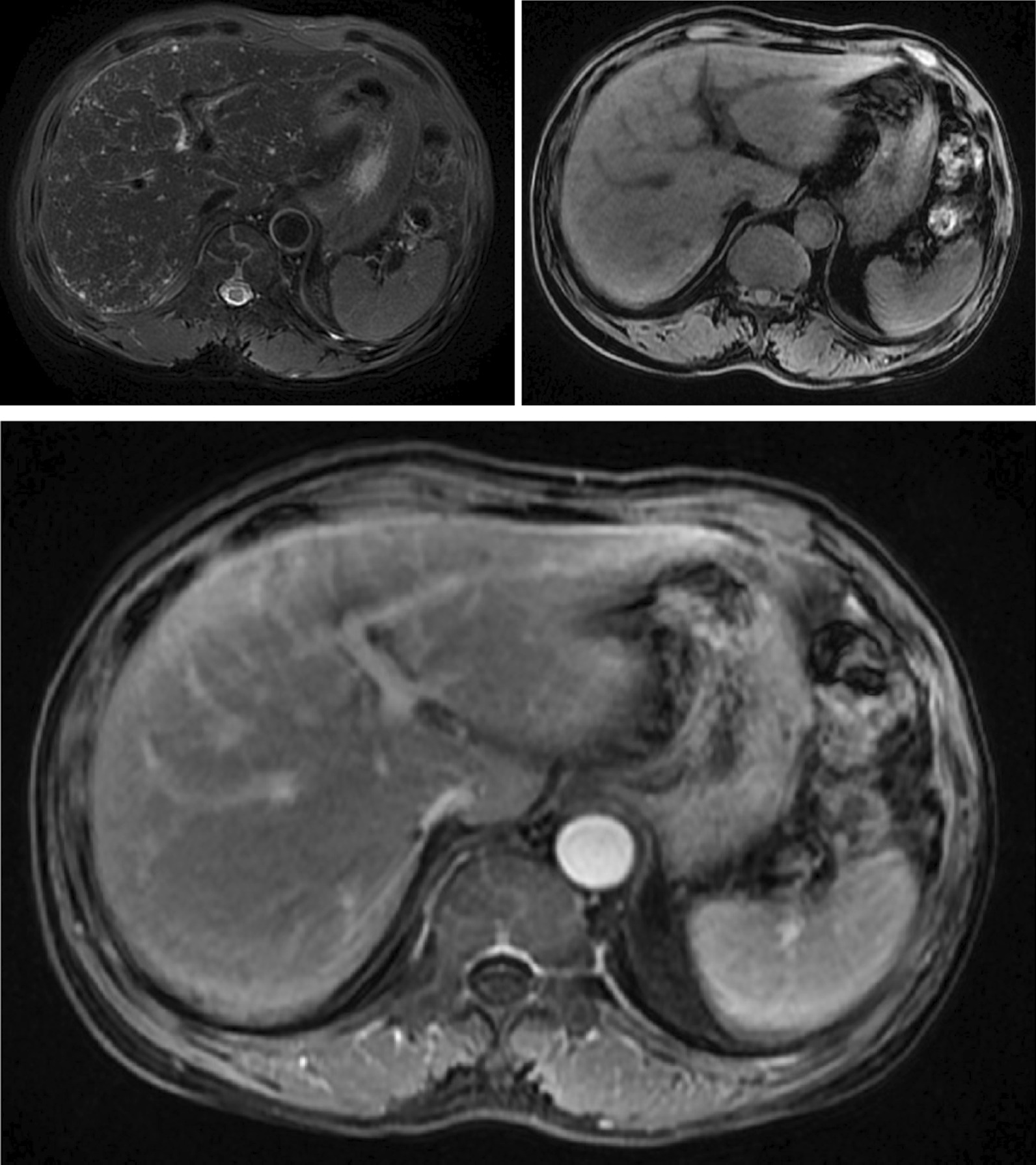
Enhanced magnetic resonance imaging of the liver. The liver images before and after contrast injection showed diffuse intrahepatic lesions

The patient was then admitted to our hospital. He complained that sensory symptoms of numbness were more prominent than his motor weakness. Physical examination revealed bilateral periphery facial paralysis, decreased temperature of the limb extremities, reduced pinprick and vibration sense, absent reflexes of both the upper and lower extremities, and an unsteady gait. Muscle strength was grade 3 in the upper and lower limbs. Nerve conduction studies showed motor and sensory demyelinating neuropathy in the upper and lower limbs (Table 1). Deep venous thrombosis was found in the right lower extremities by ultrasonography. Whole body positron emission tomography (PET)-CT scan showed diffuse low-density nodules in the liver without high glucose metabolism, which probably indicated benign lesions, bilateral renal cysts, and spinal degeneration. Enhanced MRI of the liver showed diffuse intrahepatic lesions, which were considered bile duct hamartomas in the liver (Fig. 1).

**Table 1:** Selected nerve conduction study performed at the time of diagnosis in our hospital

	Onset latency (ms)	Amplitude (mV)	Conduction velocity (m/s)
Motor nerves			
Right median nerve (wrist)	7.01	2.74	23.3
Right median nerve (elbow)	15.9	2.62	21.2
Right ulnar nerve (wrist)	5.29	1.91	Too low to calculate
Right ulnar nerve (elbow)	14.4	2.94	23.1
Right tibial nerve (knee)	No response	No response	Too low to calculate
Right common peroneal (knee)	14.3	0.052	Too low to calculate
Sensory nerves			
Right median nerve	9.08	1.4	13.2
Right ulnar nerve	4.2	2.7	23.8
Right sural nerve	No response	No response	No response
Right superior peroneal nerve	No response	No response	No response
F-wave			
Right ulnar nerve	No response		
Right tibial nerve	No response		

As the lesions were bilateral symmetry, we selected the right limbs

**Fig. 2:**
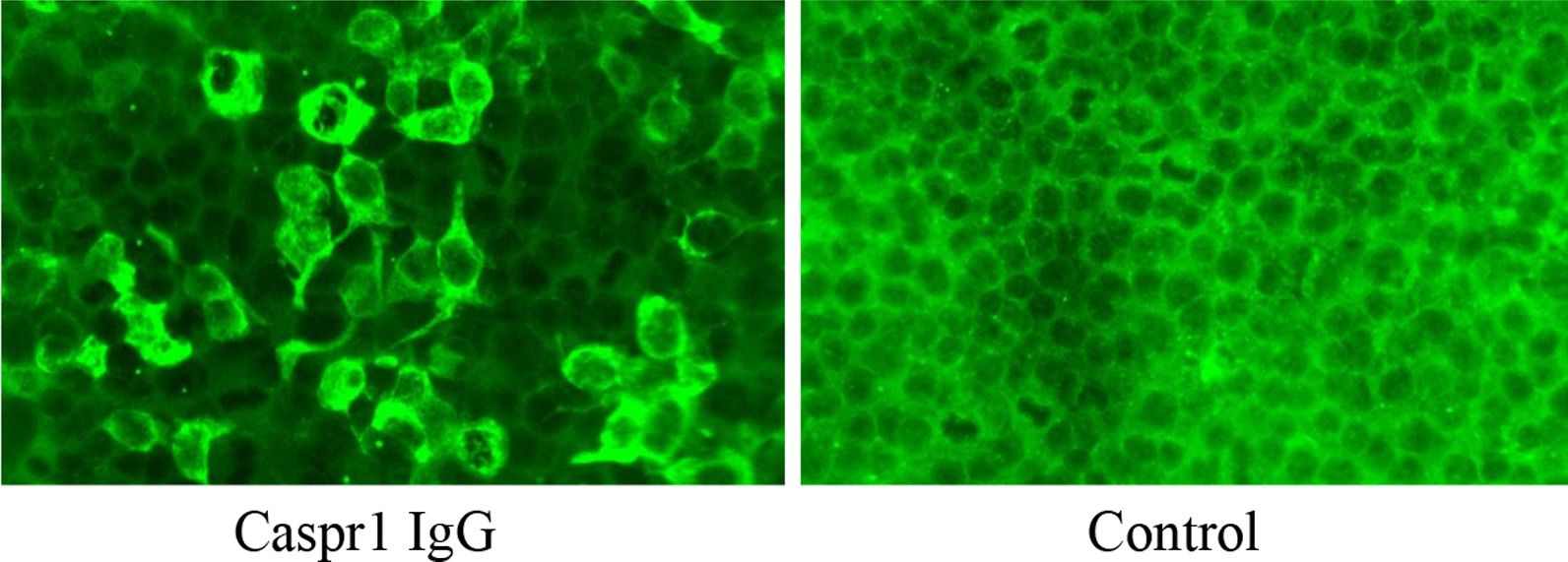
Detection of anti-Caspr1 antibody in our patient with CIDP. Serum sample from our patient was tested for auto antibodies against Caspr1 using a cell-based binding assay

The patient was diagnosed as definite CIDP, referring to the diagnostic criteria of European Federation of Neurological Societies/Peripheral Nerve Society (EFNS/PNS) [8]. The patient’s lack of response to intravenous immunoglobulins prompted us to consider autoantibodies targeting node of Ranvier proteins. Therefore, the serum sample was tested for auto antibodies against Ranvier proteins, including NF155, NF186, CNTN1, CNTN2, and Caspr1 [9]. Finally, the antibody test was positive for anti-Caspr1 antibodies (1:1000) (Fig. 2). We made the diagnosis of CIDP associated with anti-Caspr1 antibody. Then the patient received high-dose methylprednisolone, followed by standard plasma exchange and rituximab therapy. The patient’s sensory and motor manifestations were significantly improved at 1 year follow-up.

## Discussion

We report herein a patient with CIDP serology positive for anti-Caspr1 antibody in China. Worldwide, the frequency and symptoms of patients with auto antibodies against the nodal/paranodal proteins indicate significant variability between different countries, which may be caused by the various methods used for antibody detection [2]. Up to now, only a few patients have been reported with anti-Caspr1 antibodies, validating the rare presence in CIDP [10]. Caspr1 antibodies were first found in a CIDP patient with severe pain [11]. Apart from that, clinical features were quite similar to those of patients with other paranodal antibodies [12]. However, our patient did not have neuropathic pain, which is similar to the cases reported from Italy [12]. Another study showed that two patients with immunoglobulin (Ig) G4 anti Caspr1 antibodies had cranial nerve involvement and respiratory failure [7]. In a large cohort of subjects with possible CIDP, the antibodies against the node of Ranvier proteins were detected in less than 2% of patients [7]. However, antibodies against Caspr1 have only been reported in less than ten CIDP patients, and larger studies are needed to ascertain the specific features associated with these antibodies [7, 11, 12].

Anti-Caspr1 IgG4 had a function-blocking activity that inhibited the interaction of CNTN1/Caspr1- and NF155-expressing cells and enabled these auto antibodies to dismantle the axoglial interactions and penetrate the paranodal regions [12]. Intravenous immunoglobulin has been established as the first choice of treatment for CIDP. However, CIDP associated with IgG4 antibodies against the node of Ranvier proteins did not respond to intravenous immunoglobulin therapy. Due to the limited methods, it was difficult to differentiate the IgG subtype in our hospital: we suspected the antibody was IgG4 subtype because of our patient’s lack of response to intravenous immunoglobulin therapy. Plasma exchange and steroid pulse therapy were performed when intravenous immunoglobulin was not sufficiently effective. Nevertheless, rituximab treatment was needed to achieve clinical stability and the disappearance of the autoantibodies. In addition, our patient was found with bile duct hamartomas in the liver, which is rare and a type of ductal plate malformation [13, 14]. The pathogenesis of the lesions might be associated with ischemia, inflammation, or genetic anomalies [13, 14]. No relevant literature has been reported on bile duct hamartomas in liver and anti-Caspr1 CIDP. It is well known that nearly half of females presenting with anti-N-methyl-d-aspartic acid (NMDA) receptor (NMDAR) encephalitis had associated ovarian teratoma [15, 16]. Furthermore, almost all of them had positive anti-NMDAR antibody [15, 16]. Therefore, in our case, we suspected there were possible associations between bile duct hamartomas in liver and anti-Caspr1 CIDP. Further studies are needed to validate the correlation.

## Conclusions

It appears advisable to test for antibodies against node of Ranvier proteins in subjects with CIDP resistant to intravenous immunoglobulin. Although the frequency of these antibodies are rare, they have important diagnostic value and therapeutic implications as this condition does not usually respond to intravenous immunoglobulin, but may improve with plasma exchange and rituximab treatment.

## Data Availability

The data underlying this article will be shared on reasonable request to the corresponding author.

## Abbreviations

CIDP: Chronic inflammatory demyelinating polyneuropathy
Caspr1: Anti-contactin-associated protein 1
CNTN1: Contactin-1
CNTN2: Contactin-2
NF155: Neurofascin 155
NF186: Neurofascin 186
MRI: Magnetic resonance imaging
ANCA: Antineutrophil cytoplasmic antibody
ANA: Antinuclear antibody
MBP: Myelin basic protein
MOG: Myelin oligodendrocyte glycoprotein
AQP4: Aquaporin-4
PNS: Paraneoplastic neurological syndromes
